# Supplementary material for: An analytic theory for the degree of Arctic Amplification
Source: Nat Commun. 2024 Jun 13;15:5060. doi: 10.1038/s41467-024-48469-w (PMC11176348; doi:10.1038/s41467-024-48469-w)
Supplement: Supplementary file 1 — Supplementary Information [file 41467_2024_48469_MOESM1_ESM.pdf]

Supplementary Information for

An analytic theory for the degree of Arctic Amplification

Wenyu Zhou[1,*], L. Ruby Leung[1], Shang-Ping Xie[2], and Jian Lu[1]

[1] Atmospheric, Climate and Earth Sciences Division, Pacific Northwest National Laboratory

[2] Scripps Institution of Oceanography, University of California San Diego, La Jolla, California

*Corresponding Author: wenyu.zhou@pnnl.gov

Supplementary Table 1

Supplementary Figures 1-6

Model Number	Model Name	Historical	SSP2-4.5	SSP1-2.6	SSP5-8.5
**1**	ACCESS-CM2	r1-10	r1-5	r1-5	r1-10
**2**	ACCESS-ESM1-5	r1-40	r1-10	r1-10	r1-10
**3**	AWI-CM-1-1-MR	r1-5	r1	r1	r1
**4**	BCC-CSM2-MR	r1-3	r1	r1	r1
**5**	CAMS-CSM1	r1-2	r1-2	r1-2	r1-2
**6**	CAS-ESM2	r1-4	r1,3	r1,3	r1,3
**7**	CESM2	r1-10	r4,10,11	r4,10,11	r4,10,11
**8**	CIESM	r1-3	r1	r1	r1
**9**	CMCC-CM2-SR5	r1	r1	r1	r1
**10**	CNRM-CM6	r1-28	r1-6	r1-6	r1-6
**11**	CNRM-ESM2	r1-10	r1-10	r1-5	r1-5
**12**	CanESM5	r1-25	r1-10	r1-10	r1-10
**13**	EC-Earth3	r1-10	r1-7,9,10	r1-6,8,9,11,13	r1,3,4,6,9,11,13
**14**	EC-Earth3-Veg	r1-10	r1-6	r1-4,6	r1-4,6,10
**15**	FGOALS-g3	r1-6	r1-4	r1-4	r1-4
**16**	GFDL-ESM4	r1-3	r1-3	r1	r1
**17**	GISS-E2-1-G	r1-10	r1-10	r1-5	r1-5
**18**	GISS-E2-1-H	r1-5	r1-5	r1-5	r1-5
**19**	HadGEM3-GC31-LL	r1-5	r1-5	r1	r1-4
**20**	IITM-ESM	r1	r1	r1	r1
**21**	INM-CM4-8	r1	r1	r1	r1
**22**	INM-CM5	r1-8	r1	r1	r1
**23**	IPSL-CM6A-LR	r1-33	r1-6	r1-6	r1-6
**24**	KACE-1-0-G	r1-3	r1-3	r1-3	r1-3
**25**	MIROC-ES2L	r1-10	r1-10	r1-10	r1-10
**26**	MIROC6	r1-50	r1-10	r1-10	r1-10
**27**	MPI-ESM1-2-LR	r1-10	r1-10	r1-10	r1-10
**28**	MPI-ESM1-2-HR	r1-10	r1-2	r1-2	r1-2
**29**	MRI-ESM2	r1-10	r1-5	r1-5	r1-5
**30**	NESM3	r1-5	r1-2	r1-2	r1-2
**31**	NorESM2-MM	r1-3	r1,2	r1	r1
**32**	UKESM1-0-LL	r1-19	r1-4,8,13	r1-10	r1-4,8

Table S1: The CMIP6 models and their realizations used in this study for the historical and SSP experiments.

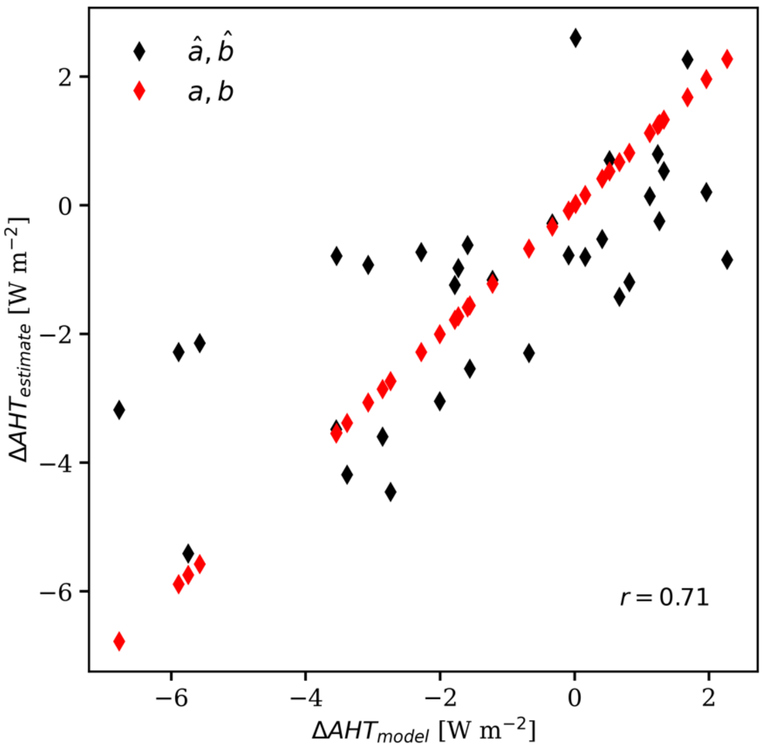

Figure S1: The change in atmospheric heat transport into the Arctic simulated by models (ΔAHT_{model}) versus that estimated as a function of changes in global and Arctic mean surface temperature ($\Delta AHT_{estimate}$; Eq. 5) using constant $\hat{a}$ and $\hat{b}$ (black) and model-dependent a and b (red).

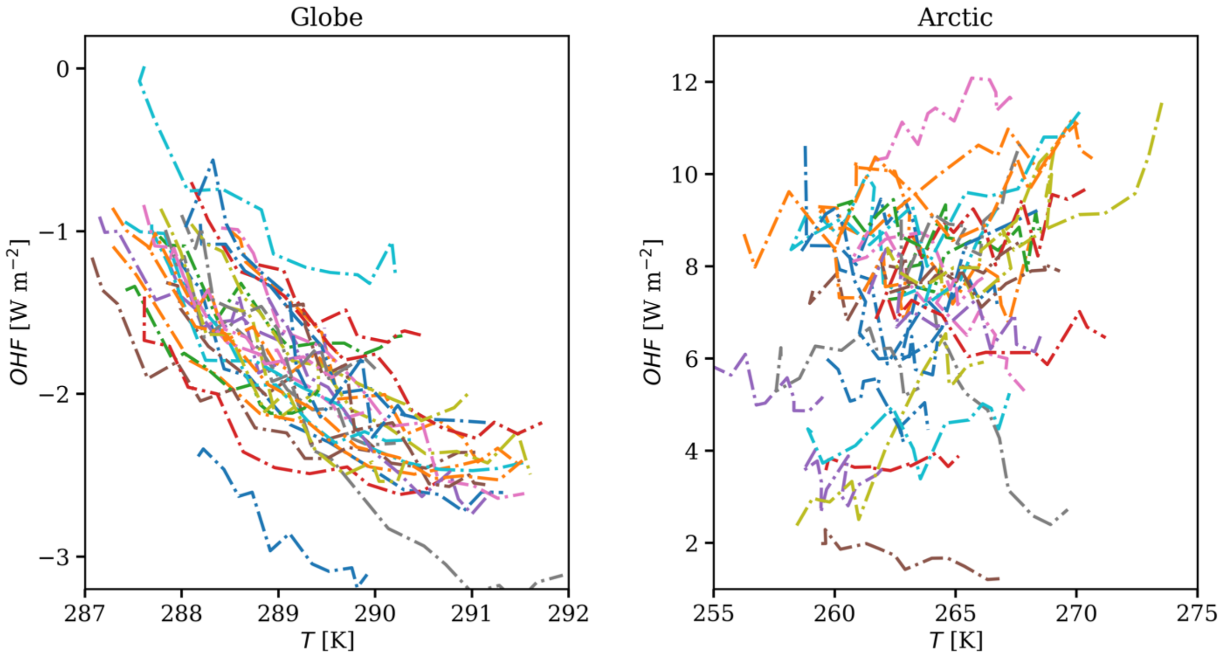

Figure S2: Changes in ocean heat convergence and uptake, ΔO, as a function of temperature for the global and the Arctic. Each line represents a model.

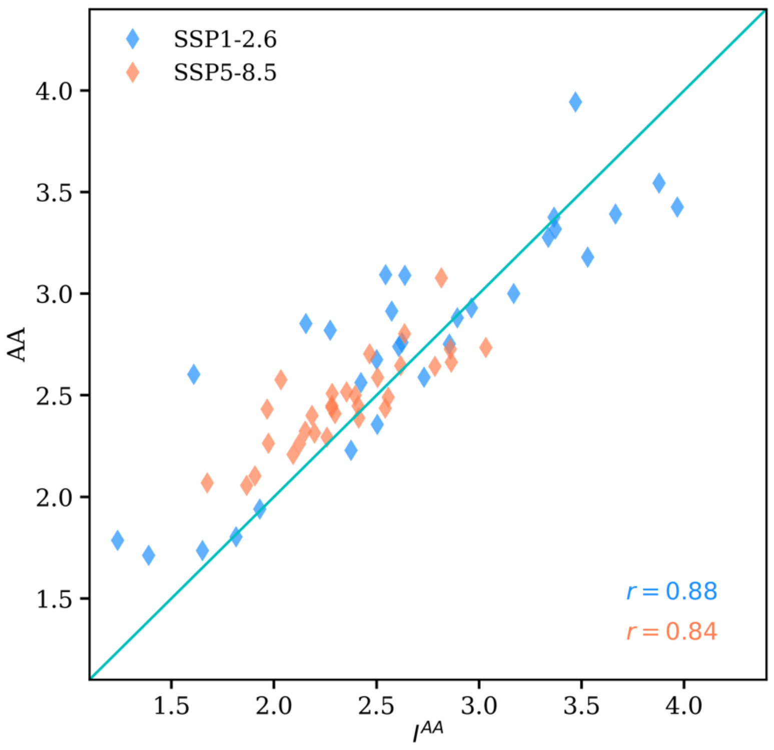

Figure S3: Scatterplot between the model-projected AA and the theoretical estimate ($I^{AA} \equiv 1 + \frac{a+\lambda^A - \hat{\gamma}\lambda^G}{b-\lambda^A}$) for the low (SSP1-2.6) and high (SSP5-8.5) emission scenarios.

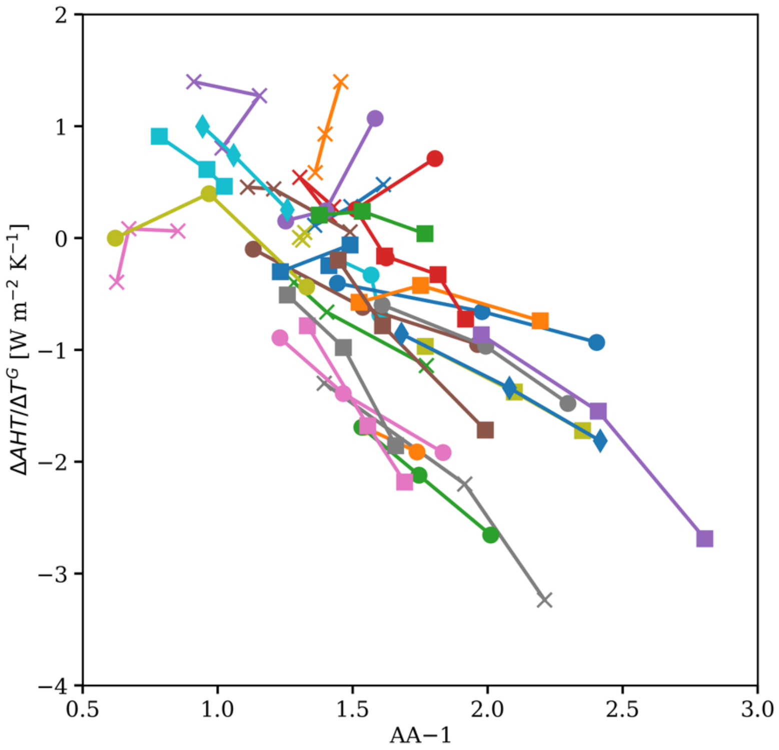

Figure S4: Scatterplot between AA-1 and the change in atmospheric heat transport into the Arctic normalized by the global mean warming ($\Delta \boldsymbol{AHT}/\Delta \boldsymbol{T^G}$) across models. The SSP126, SSP245 and SSP585 warming scenarios are considered and show in symbols. The results of SSPs of each model is connected by a line.

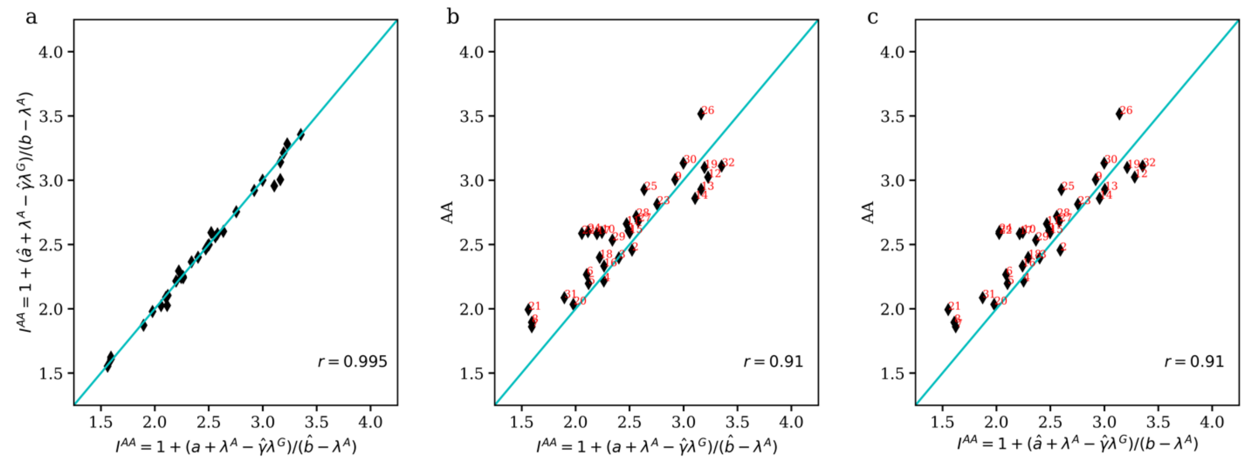

Figure S5: a, Scatterplot between the theoretical estimates with the difference between the model-projected $\frac{\Delta AHT^A}{\Delta T^G}$ and the prior guess $\widetilde{\frac{\Delta AHT^A}{\Delta T^G}} = \hat{a} - \hat{b}(AA-1)$ attributed all to a ($I^{AA} = 1 + \frac{a+\lambda^A-\hat{\gamma}\lambda^G}{\hat{b}-\lambda^A}$; x axis) or b ($I^{AA} = 1 + \frac{\hat{a}+\lambda^A-\hat{\gamma}\lambda^G}{b-\lambda^A}$; y axis). b, Scatterplot between the model-projected AA and $I^{AA} = 1 + \frac{a+\lambda^A-\hat{\gamma}\lambda^G}{\hat{b}-\lambda^A}$. c, Scatterplot between the model-projected AA and $I^{AA} = 1 + \frac{\hat{a}+\lambda^A-\hat{\gamma}\lambda^G}{b-\lambda^A}$.

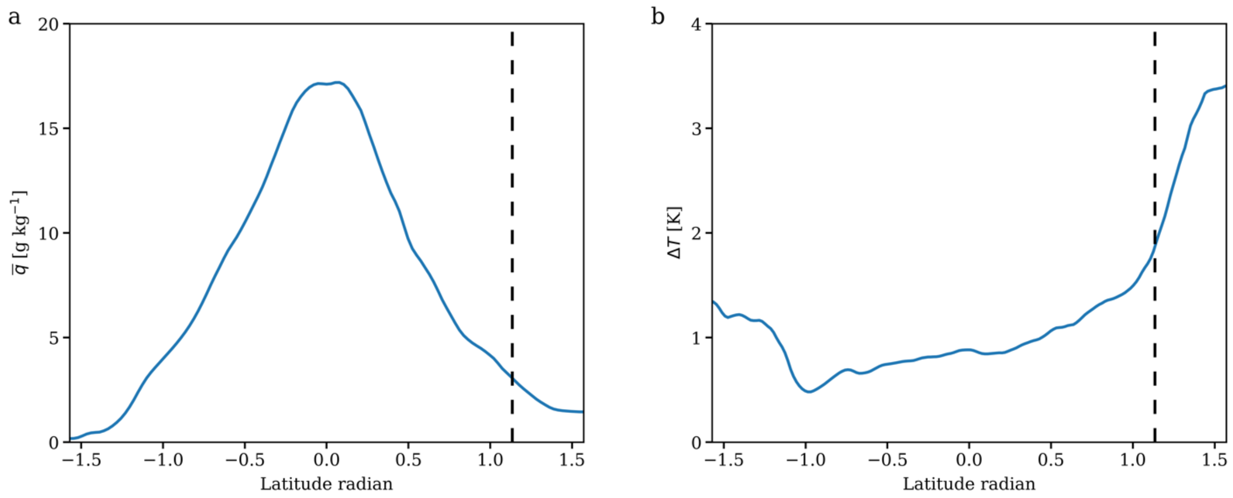

Figure S6: a, Zonal-mean annual-mean of the climatological surface (2m) specific humidity as a function of latitude radian. b, Zonal-mean annual-mean of surface warming as a function of latitude radian under SSP2-4.5. The latitude of 65°N is denoted by the dashed line.
